# From Retrofitting to Adaptive Fitting: Time Scale and Locus of Change Capacity in Adaptive and Demountable Building Envelopes

**DOI:** 10.3390/biomimetics11100746

**Published:** 2026-10-16

**Authors:** Zelal Çınar

**Affiliations:** Department of Architecture, TOBB University of Economics and Technology, 06560 Ankara, Türkiye; zcinar@etu.edu.tr

**Keywords:** biomimetics, building envelope, adaptive façade, design for disassembly, adaptation, retrofit, life cycle, design for adaptability

## Abstract

Building envelopes are addressed by two largely separate bodies of research: adaptive façade design, which treats change as behaviour during operation, and design for disassembly, which treats it as an end-of-life property. This study asks whether that separation reflects a division in the problem or in academic labour. Four modes of intervention through which envelopes are kept fit (add-on, replace, cover it, and wrap it) are mapped onto four fitting modes of natural skin; the correspondence weakens as the intervention becomes more external. Fifteen documented cases are then coded against a scheme separating the time scale at which change is accommodated from the location and type of the capacity accommodating it, with predictions recorded before coding. Every biomimetic case operates at daily or seasonal scale, and fourteen of fifteen cases return a functioning envelope when their adaptive capacity is removed. Cases addressing life-cycle change do so through external agency, and none has undergone the change it anticipates. No case addresses the intermediate scale of years to decades, at which buildings in fact become unfit. The unoccupied combination is termed adaptive fitting here, and the argument advanced is that the obstacle is evaluation before capability: envelopes are assessed on how they behave, not on how long they continue to behave that way.

## 1. Introduction

Buildings cease to fit. Requirements move, occupancies change, components reach the end of their service life, and the climate a building was designed for is no longer the climate it stands in. The response is retrofit: an intervention applied once the building has already become unfit, and applied from outside, by parties other than the building itself. Retrofit is necessary and will remain so for the existing stock. The question this paper addresses is whether it is the only available model for how a building remains fit, and specifically whether the building envelope, as the element that mediates most of the change a building experiences, can be designed to accommodate change rather than to receive it.

Two bodies of work bear on this question, and they have developed largely independently.

### 1.1. Adaptive and Biomimetic Building Envelopes

The first treats the envelope as a dynamic system. The idea has a long lineage in architectural writing on intelligent and interactive envelopes [1,2]. Since the survey of climate-adaptive building shells by Loonen et al. [3,4], research on envelopes that modify their behaviour in response to environmental conditions has grown into an established field with its own reviews, taxonomies, and design frameworks. A substantial part of it is explicitly biomimetic. Badarnah [5,6] developed methods for generating envelope design concepts from biological principles. Kuru et al. [7,8,9] defined biomimetic adaptive building skins as a category, characterised the existing corpus, and proposed a framework for achieving multifunctionality, reporting that a small minority of documented examples achieve it. Finally, Sommese et al. [10] surveyed the field critically and proposed a bio-adaptive model. The best-known instances range from the mechanical to the material. These extend from the motorised mashrabiya-inspired diaphragms of the Institut du Monde Arabe (1987) and the folding shading screen of the Al Bahar Towers (2012), both of which appear in the case set below, through to Solar Gate, a hygroscopic shading system installed on a permanent research building that operates on daily and seasonal weather cycles without electrical supply, sensors or controls [11]. Others continue to develop the field computationally or at model scale [12].

Across this body of work, adaptation is understood as behaviour during operation. The changes addressed occur over hours, days, and seasons; the performance criteria are thermal, visual, and energetic; and the assessment methods—simulation, prototype testing, monitored field trials—are suited to those durations.

### 1.2. Adaptability over the Building Life Cycle

The second body of work treats change as a property of the building’s life rather than of its operation. Brand’s account of shearing layers [13] established the durable observation that a building’s components change at different rates and that design should reflect this. The concern has since been formalised: ISO 20887 [14] sets out principles for design for disassembly and adaptability, ISO 52016-3 [15] provides calculation procedures for adaptive envelope elements, and circular economy policy has made reuse and disassembly explicit objectives. Buildings have been delivered on these principles, including the Triodos Bank headquarters, assembled without adhesives and documented in a material passport, and the Circle House housing scheme, reduced to a small number of repeating demountable elements.

Here, adaptation means the capacity to be taken apart and reconfigured, and the agent is external: a contractor, a facilities team, or a future designer. The provision is a design property, exercised by others at a later date. Hartwell et al. [16] note that disassembly of contemporary façade systems is not standard end-of-life practice; instead, demolition remains the norm. A body of work has since developed assessment methods for the reclamation potential of façade systems [17,18,19], alongside earlier proposals for reversible building design [20].

### 1.3. The Disconnection Between Them

That these two literatures have little contact has been observed. Niazy et al. [21] identified the absence of circularity considerations in adaptive façade design and proposed a module combining an operational adaptive capacity with a demountable one, framing the problem as one of end-of-life material recovery. The framing adopted here is different. The question is not only what becomes of an adaptive envelope when the building is demolished. It is where the capacity for change is located and for how long it operates, and whether the separation between the two literatures reflects a real division in the problem or only a division of academic labour.

**Figure 1:**
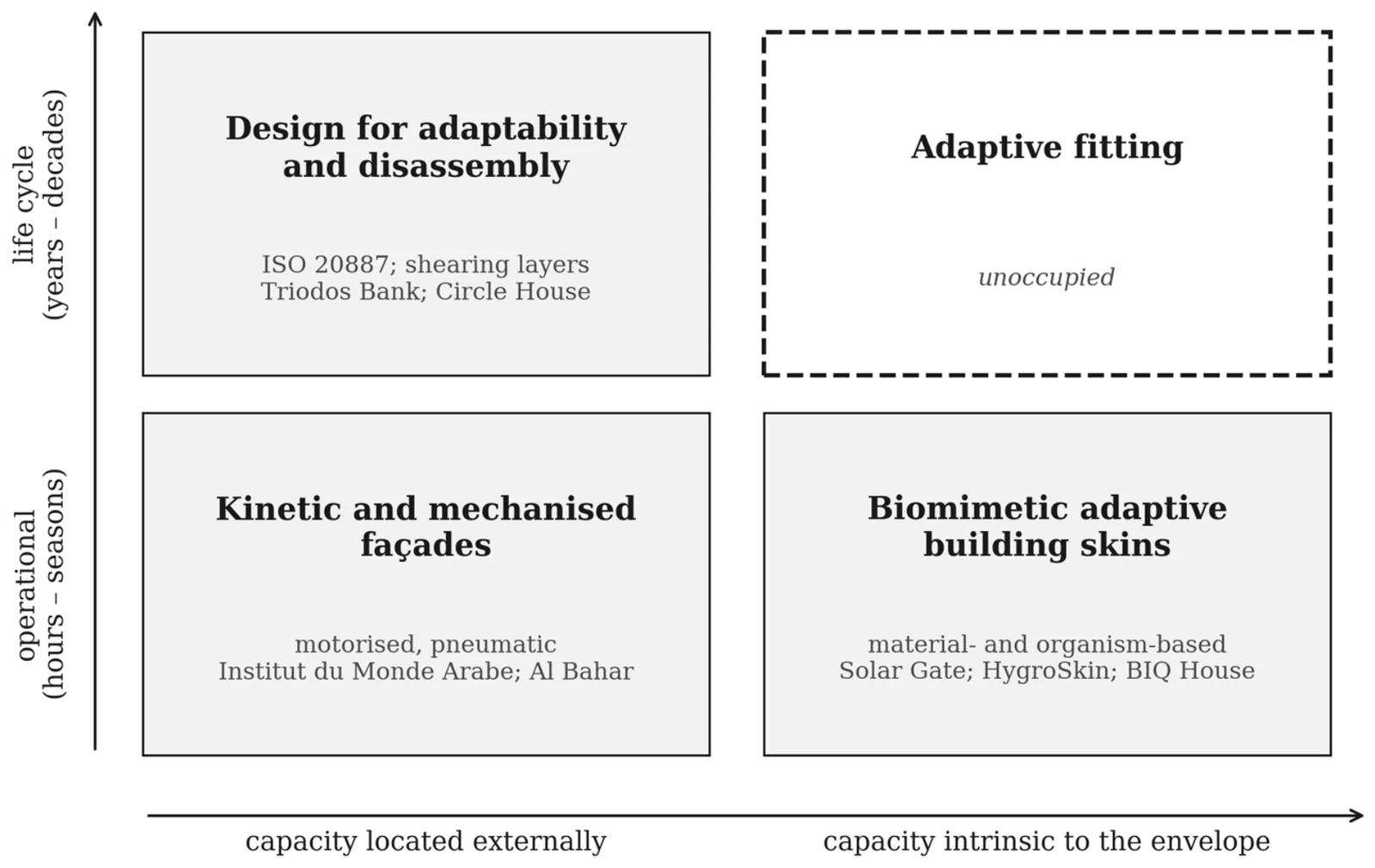
Positioning of the two bodies of work on two axes: the time scale over which change is accommodated, and the location of the capacity that accommodates it. The combination of an integrated, autonomous capacity with a life-cycle time scale is unoccupied.

There is a related but distinct concern in recent biomimetic work. Varshabi et al. [22] observed that organisms regulate heat, light, air, and moisture through a single integrated organisation, while architecture addresses these functions separately, and they proposed an integrated framework in response. That is a claim about functional integration within the operational scale. The claim examined here concerns temporal integration: whether the same organisation that serves a building’s daily behaviour can also serve its long-term fitness. Figure 1 sets out the two axes on which the two bodies of work are positioned in what follows.

### 1.4. Why the Analogy

Natural skin is the only available model in which both scales are served by the same means. Skin regulates temperature and light over the course of a day; it also regenerates, grows, thickens, and is periodically renewed over the life of the organism. These are not separate systems. The capacity for both resides in the same tissue, and it is present before the need arises rather than supplied when the need is recognised. Where architecture separates operational adaptation from life-cycle adaptability, and locates the second outside the building, biology does neither [23].

This is the sense in which the present study is biomimetic. It does not propose to imitate a mechanism. It uses the organisation of natural skin as a standard against which the organisation of building envelopes can be described in order to make visible a distinction that architectural practice does not currently draw.

One term requires clarification before it is used further. In its biological sense, regeneration appears below as one of four fitting modes of natural skin: the repair or replacement of tissue by the organism itself. It is not used in the sense it carries in regenerative design, where it denotes a building’s net positive contribution to the ecological systems it sits within. The two share a word and a concern with restoration, but not a referent. The argument developed here is about where the capacity for change is located and for how long it operates, and it is compatible with regenerative design without being an instance of it.

### 1.5. This Study

This study asks three questions. What are the modes by which building envelopes are currently kept fit, and how do they correspond to the fitting modes of natural skin? Over what time scales, and by what located capacity, do contemporary adaptive and demountable envelopes operate? And what design approach follows from the pattern that emerges?

Section 2 sets out the analogical approach, the case set, and the coding scheme, together with predictions recorded before coding. Section 3 identifies four modes of intervention through which envelopes are currently kept fit, maps them to the four fitting modes of natural skin established in earlier work, and reports the coding of fifteen cases. Section 4 derives from these results a design approach, termed adaptive fitting, examines why the combination it describes has not been attempted, and states the limits of both the evidence and the proposition. Section 5 presents the conclusions.

The principal contributions are three. First, a mapping between the modes by which envelopes are intervened upon and the modes by which natural skin maintains fitness, which shows the correspondence weakening precisely as the intervention becomes more external. Second, a coding scheme that separates the time scale at which change is accommodated from the location and type of the capacity accommodating it, applied to fifteen documented cases. The coding finds that a material-based, autonomous capacity has been achieved in buildings but only at operational scale, that provision for life-cycle change is uniformly external, and that no case combines the two. Third, an account, advanced as a hypothesis, of why that combination remains unattempted, which locates the likely obstacle not in the capabilities of the field but in the criteria by which envelopes are evaluated: an adaptive envelope is assessed on how it behaves, not on how long it continues to behave that way.

## 2. Materials and Methods

### 2.1. Approach

Biomimetics has no single established method for transferring knowledge between biology and design, and the appropriate procedure is generally taken to require reconsideration for each design problem. The approach used here is pattern-based [24]: strategies observed in one domain are abstracted to the level at which they can be compared with strategies in the other, and the comparison is then used to identify what is present in one domain and absent from the other. The four fitting modes are used here as a working taxonomy rather than a claim about biological mechanism. Their status and the analogical rather than deductive character of the mapping are discussed in Section 4.3.

The biological side of the comparison is not developed anew in this paper. Four fitting modes of natural skin (regeneration, growth, protection, and communication) were established through a survey of adaptation strategies across taxa in the author’s doctoral research and in a conference paper co-authored with their supervisor [25,26], and they are used here as a given. That work also proposed an initial mapping between these modes and the functions of the building envelope. The present study extends it in three respects: it identifies the modes of intervention through which envelopes are currently kept fit and maps these to the natural modes (Section 3.2); it introduces time scale and locus of capacity as coding variables and applies them to a case set (Section 2.3 and Section 3.4); and it derives a design approach from the resulting pattern (Section 4.1).

To make the relationship to that earlier work explicit, its contribution and the contribution of the present study can be separated as follows. From the doctoral research and the conference paper [25,26] come the four fitting modes of natural skin used in Section 3.1 and an initial mapping between those modes and the functions of the building envelope. New to the present study are the four modes of intervention through which envelopes are kept fit and their mapping onto the natural modes (Section 3.2); the five coding variables introduced here (change time scale, recorded as intended and observed; removability; capacity type; agency; and validation status; Section 2.3); the enlarged and recoded set of fifteen cases, of which eight are carried over from the earlier work and recoded against the extended scheme; and the design approach, adaptive fitting, derived from the resulting pattern (Section 4.1). The earlier work established the biological vocabulary; the analysis, the coding scheme, the case set, and the argument built on them are developed here.

The study proceeds in two stages. The first is analytical: the interventions applied to existing envelopes are examined for their underlying principle and compared with the natural modes. The second is empirical: a set of documented envelope cases is coded against a scheme that separates the time scale at which change is accommodated from the location of the capacity that accommodates it.

### 2.2. Case Selection

Fifteen cases were selected; they are listed with their codings in Section 3.4. The set is purposive rather than systematic. Cases were chosen as prominent, adequately documented instances of the approaches under comparison, rather than sampled to represent the distribution of practice.

The selection follows a strongest-case logic rather than a representative one. The cases are among the most visible and best-documented instances of each approach: Solar Gate is published in Nature Communications, the BUGA Wood Pavilion is a flagship of its research programme, and the adaptive façades of Group A are the canonical examples cited across the field. The reasoning is that if a combination is absent even from the most advanced and most fully reported work, its absence is unlikely to be an artefact of overlooking marginal projects. What follows from the selection for the status of the central finding—a result for this set rather than a survey of the field—is set out in Section 4.3.

The set is built in two parts, with different inclusion criteria that the analysis then compares. The primary cases (Groups A and B) were included when the adaptive or change-accommodating provision is located in the building envelope. A smaller comparison set (Group C) was included on the opposite basis—projects whose provision for long-term change is located in the structure or in whole-building demountability rather than in the envelope. The comparison group is essential to the argument rather than incidental to it. The claim advanced here is precisely that provision for life-cycle change is currently made in the structure and by external disassembly, not in the envelope’s own organisation, and Group C is the evidence for the first half of that contrast. Both parts were included only when the case is documented in a peer-reviewed publication or by primary institutional sources sufficient to support coding on all eight variables and when the provision is intended to operate during the building’s life rather than only during its fabrication.

The third condition excludes a class of cases that would otherwise qualify. Several recent projects use hygroscopic self-shaping to form components (curved timber shells produced by controlled drying, for instance), where the material’s responsiveness is used during manufacture and is not active thereafter. These are relevant to biomimetic fabrication but not to the question examined here.

The cases fall into four groups.

Group A (eight cases) consists of adaptive envelopes documented in the earlier study [26], recoded here against the extended scheme: Institut du Monde Arabe [27], Al Bahar Towers [27,28], Media-TIC [27,29], Kunsthaus Graz [30], BIQ House [31], Homeostatic Façade System [32], HygroSkin [33,34], and Flectofin [35]. Four cases from that study were omitted as redundant with the cases retained: two duplicated the coding profile of Institut du Monde Arabe and Kunsthaus Graz respectively, one shared a mechanism family with Flectofin, and one was a single dwelling with limited documentation.

Group B (three cases) consists of biomimetic envelopes published after 2018 whose adaptation is operational: Solar Gate at the livMatS Biomimetic Shell [11], the FlectoLine façade demonstrator [36,37], and the Mimosa-inspired kinetic façade of Sankaewthong et al. [12].

Group C (three cases) consists of projects whose change is addressed at the life-cycle scale through disassembly and component reuse rather than through operational adaptation: the BUGA Wood Pavilion [38], the Triodos Bank headquarters [39], and Circle House [40]. The BUGA Pavilion is grouped here, rather than with the biomimetic envelopes of Group B, because its provision for change is a life-cycle one—the segmented timber shell is designed to be dismantled and its components reused—which is the behaviour that defines this group, even though the pavilion is itself a biomimetic structure. The Circle House residential development has not been confirmed as completed, and the case is therefore treated as a pilot project for circular design strategies rather than as a completed building; the demonstrator unit built in 2020 is what the coding reflects.

Group D (one case) consists of the single identified attempt to combine the two approaches: the circular adaptive façade module of Niazy et al. [21].

Two limitations of the set follow from its construction and are discussed in Section 4.3. Group B is concentrated in a small number of research programmes, and Group C is a small comparison set assembled to illustrate structural, rather than envelope-based, provision for change, not to sample it exhaustively.

### 2.3. Coding Scheme

Eight variables were coded (Table 1). Three—scale of change, fitting mode, and stimulus source—are adapted from the scheme used in the earlier study [26]; five are introduced here: change time scale (recorded as both an intended and an observed value), removability, capacity type, agency, and validation status. The change from the earlier study is not only the number of variables but the separation of dimensions that a single “integration” variable had previously combined—removability (whether the provision can be taken out) and agency (what exercises the capacity) are now coded independently, as are the intended and observed time scales.

Scale of change (retained). WE, the whole envelope as one piece; PE, parts or volumes within the envelope; LEE, a larger element; SEE, small elements.

Fitting mode (retained). R, regeneration; G, growth; P, protection; C, communication.

Stimulus source (retained, redefined). E, an environmental (external) stimulus such as light, heat, humidity or wind; I, an internal stimulus such as a building-system signal or schedule. This variable records the source that triggers the response, not the location of the responsive capacity. For example, a hygroscopic element whose capacity lies in the material but which responds to ambient humidity is coded E, because the trigger is environmental. Where a case has no operational response (the life-cycle cases of Group C), the variable is not applicable.

Change time scale (new). Intended: T1, seconds to days; T2, weeks to seasons; T3, years to decades; T4, decades to end of life. This variable records the scale the design is intended to accommodate. Observed: the scale at which a change or failure has actually been documented, left empty where none is reported. A case may be coded at more than one intended scale.

Removability (new). INT, removing the adaptive capacity leaves no functioning envelope (integrated); SUB, removing it leaves a functioning envelope (substitutable).

Removability is applied by a single test: whether an envelope stripped of its change-accommodating provision would still perform as an envelope. The test was chosen for consistency of application rather than because it resolves every case cleanly, and cases where it returns a result at odds with other readings are noted in Section 4.3.

Capacity type (new). MAT, capacity resides in the physical behaviour of the material; LIV, in a living medium (a cultured organism such as the microalgae of a bioreactor façade); MEC, in a discrete mechanism. Where the provision is not a material or mechanism but a disassembly protocol, the cell is left empty.

Agency (new). SELF, the capacity operates autonomously, without supplied energy; ACT, the capacity is actuated by supplied energy (a motor, pump or electrical charge); EXT, the capacity is exercised by an external agent (a contractor or facilities team).

Removability, capacity type, and agency are coded independently. A case may be material-based (capacity type MAT) and still require an external agent to exercise that capacity (agency EXT), or constitute an addition to a conventional envelope (removability SUB) while operating without any supplied energy (agency SELF).

Validation status (new). BUILT, constructed and in service; PROTO, prototype, pavilion or component not in building service; SIM, simulation only.

Three coding rules were applied. First, what has occurred is coded rather than what was designed: where a case anticipates a change that has not taken place, the intended time scale records the design intent, the observed time scale records what has actually happened (left empty where nothing is reported), and the validation status records the evidential basis of the case itself. Second, where documentation is insufficient for a variable, the cell is left empty rather than inferred. Third, stimulus source and agency were checked independently for every case since the two answer different questions: what triggers the response (environmental or internal) is not the same as what supplies the energy or effort that carries it out (autonomous, actuated or external). A case can therefore be triggered environmentally while operating without supplied energy, as HygroSkin and Solar Gate do, or triggered environmentally while depending on an actuator, as the Homeostatic Façade System does.

### 2.4. Predictions

Predictions were recorded before coding began and are reported here unchanged.

The primary prediction was that no case would combine a capacity that is integrated and either autonomous or actuated (removability INT with agency SELF or ACT) with a life-cycle intended time scale (T3 or T4). A secondary prediction was that the regeneration and growth modes would be largely absent from Groups A and B, and that where they occurred the capacity would be externally located.

Conditions under which the predictions would be treated as unsupported were also recorded. Had a case occupied that combination, a T3 or T4 intended scale coded INT with SELF or ACT agency, the claim that the intersection is unoccupied would have been withdrawn and that case would have become the study’s principal subject. Had Groups A and B proved to span a wider range of time scales than predicted, the argument that the two literatures address different time scales would have been correspondingly weakened.

Both predictions were supported. The manner in which they were supported is reported in Section 3.4 and differs in one respect from what was anticipated: an integrated, autonomous capacity was found to be achievable and achieved, but only at operational scale.

The separation of capacity from agency resolves a case that a single “material-based” category would have blurred. The Homeostatic Façade System uses a dielectric elastomer—a material that changes shape—but the change requires a supplied electrical charge and an actuator. It is therefore coded with a material capacity (MAT) and an actuated agency (ACT): what changes is the material, but what drives it is supplied energy. The same rule applies consistently to other material effects that need an energy input, such as piezoelectric or thermoelectric elements: the material locates the capacity, while the need for supplied energy places the agency under ACT rather than SELF. Only materials that respond without added energy—the hygroscopic elements of HygroSkin and Solar Gate—are coded SELF. This recoding was made before the analysis was written.

### 2.5. Use of Generative AI Tools

A generative AI assistant (Claude, Anthropic; Opus 4.8, accessed 20 July 2026) was used during the preparation of this study. Its use extended beyond language editing and is described here in full. The assistant was used to search and summarise literature published after the earlier work on this subject [25,26]; to identify candidate cases and locate documentation for them; to propose the coding variables introduced in Section 2.3 and the removal test by which removability is applied; to produce an initial coding of the case set; to draft text for all sections of this manuscript; to generate the figures from Python (version 3.11) scripts written by the assistant; and to assemble the reference list.

All outputs were reviewed, corrected, and accepted or rejected by the author. Several coding decisions produced by the assistant were revised on the author’s judgement, including the removability coding of the BIQ House and the validation status of Circle House, the latter following verification by the author against municipal and architectural office documentation that contradicted the assistant’s initial reading. Case selection was determined by the author. The predictions reported in Section 2.4 were recorded before coding and are the author’s responsibility, as is every claim, citation, and interpretation in this paper. The assistant is not an author and does not meet authorship criteria.

## 3. Results

### 3.1. Four Fitting Modes of Natural Skin

The comparison rests on four modes by which natural skin maintains fitness, which were established in earlier work through a survey of adaptation strategies across taxa and are summarised here (Table 2) [25,26,41,42,43].

Regeneration covers renewal and restoration, such as the replacement of lost or damaged tissue through the organism’s own processes, as in autotomy in lizards or renewal in sea stars. Growth covers accretion at an edge, the growth of organisms carrying shells, moulting, and addition, that is, the modes by which a covering is enlarged or exchanged as the body it encloses changes size. Protection covers strengthening and colouration, including the hardening of an outer layer against mechanical stress and seasonal changes in coat colour that alter insulating performance. Communication covers signalling and sexual attraction, in which the surface is used to transmit information rather than to regulate an exchange.

Two features of this set matter for what follows. First, the four modes operate at different durations. For example, colouration in a chameleon occurs over seconds, whereas moulting occurs over a life stage, yet they are not organised as separate systems. Second, in every case, the tissue that changes is also the agent of that change.

### 3.2. Four Modes of Intervention in the Building Envelope

When the interventions routinely applied to existing envelopes are analysed for their underlying principle rather than their technical means, four categories emerge; each is set against its natural counterpart in Table 2. Each has a counterpart among the fitting modes of natural skin. The correspondence, however, is uneven, and the unevenness is itself informative.

Add-on. A new structure is attached to an existing building when additional space, function, or appearance is required, ranging from a balcony to a full lateral or vertical extension. Where the extension is lateral, the old envelope ceases to act as an envelope and the new one absorbs its performance requirements. The natural counterpart is regeneration, where organisms rebuild lost or damaged tissue through internal processes, without demolition and without transforming the surrounding tissue. Buildings reach a comparable outcome by the opposite route. Material is brought to the site and attached to a fabric that remains passive throughout.

Replace. Envelope elements are removed and substituted while the load-bearing structure and interior layout are retained; the intervention may extend from a single component to the entire envelope. Foster and Partners’ Reichstag replaced the original cupola with a lighter and transparent one, simultaneously addressing aesthetic, accessibility, and environmental requirements. The analogue in nature is shell exchange and moulting. As hermit crabs outgrow their shells, they abandon them for larger ones, and the vacated shell is occupied by a smaller individual, so that nothing is discarded. Replacement in buildings normally entails demolition and new construction. The mode is shared; the resource logic is not.

Cover it. Courtyards, atria, or the residual space between existing buildings are roofed over, converting exterior to interior and adding usable area. The nineteenth-century iron-and-glass arcade is the early type; Foster and Partners’ Great Court at the British Museum is a contemporary instance. Growth here is achieved by enclosure rather than by extension, which alters the relationship between inside and outside without adding footprint. In nature, growth of this kind occurs through the incremental accretion of material at an edge, governed by the organism’s own developmental programme. In buildings, the enclosing element is designed and installed as a discrete addition, and the relationship it establishes is fixed at the moment of installation.

Wrap it. A second layer is applied over the existing envelope, from the cladding of balconies to the wrapping of a whole building. Gehry’s own residence in Santa Monica encased an existing house in plywood, corrugated metal, and chain-link, extending it toward the street while leaving the original envelope largely untouched. Because the operation is carried out from outside, it does not suspend the building’s use, which accounts for much of its practical appeal. It also has the weakest counterpart in nature. Metamorphosis is sometimes invoked, but in metamorphosis, transformation proceeds from within and in stages and the organism that emerges does not retain the structure it replaced. Wrapping preserves the old envelope precisely by rendering it inert.

Taken together, the four modes describe the repertoire through which envelopes are currently kept fit. They are not, however, equivalent in how they relate to their natural counterparts.

### 3.3. What the Mapping Reveals

**Figure 2:**
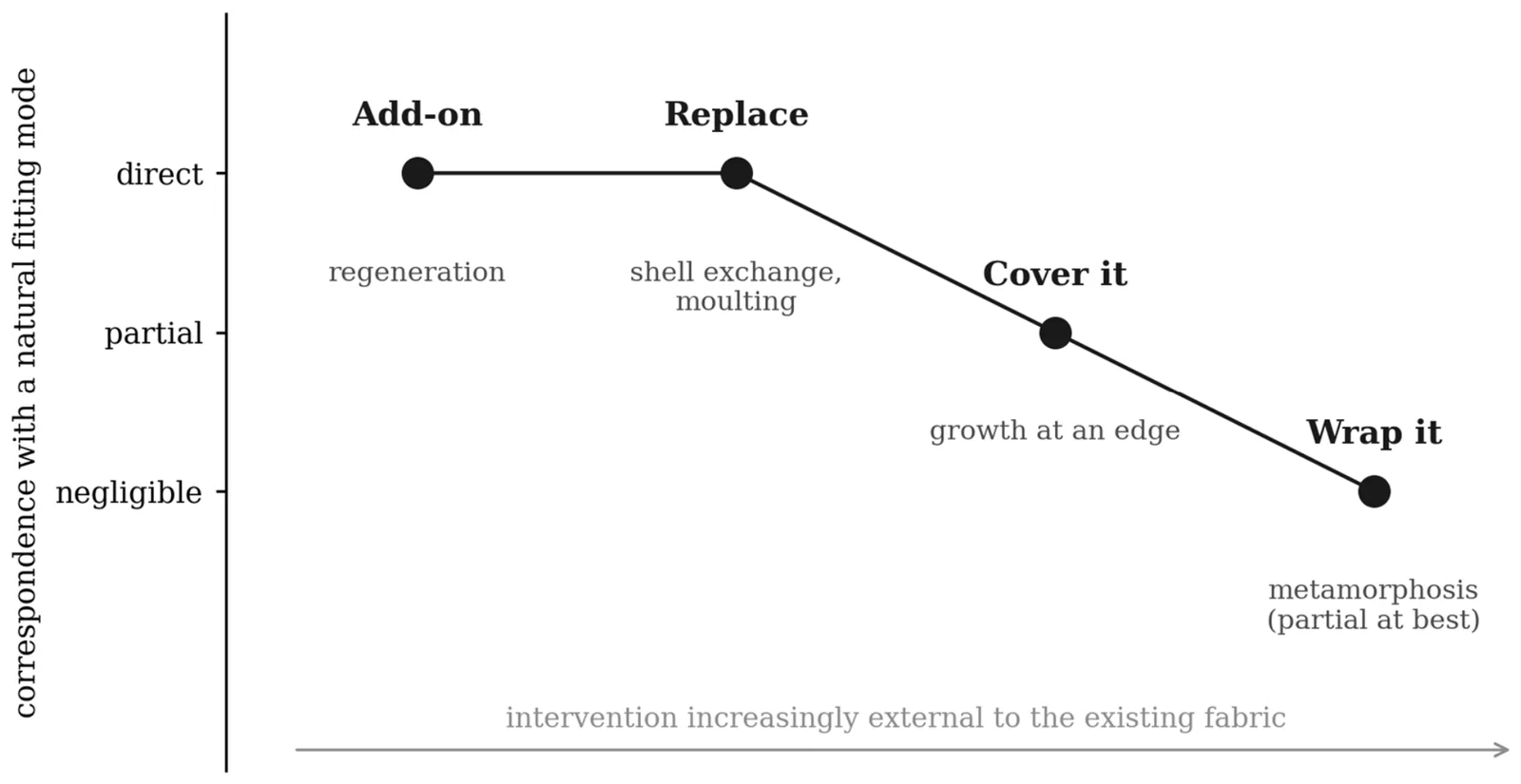
Conceptual ordering of the correspondence between each mode of intervention and its natural counterpart, arranged by how far the operation stands from the existing fabric. The vertical position is a qualitative judgement (direct, partial, negligible), not a measured quantity; the figure is a diagram of the argument rather than an analytical result.

The four modes do not stand at an equal distance from their natural counterparts. Arranged by the locus of the operation rather than by its scale, cost, or technical sophistication, they form a gradient (Figure 2). Add-on and replace, which engage the fabric of the building directly, admit reasonably direct analogues. Cover it, which acts on the space a building encloses rather than on its material, admits a partial one. Wrap it, which leaves the existing envelope in place and inert, admits almost none.

This ordering is qualitative—a conceptual summary of the mapping rather than a measured result—and Figure 2 should be interpreted as a diagram, not as data; viewed that way, the ordering is still informative. The difficulty in mapping wrapping onto a natural process is not a defect of the analogy but a reflection of the distance between the two domains at that point. Where the correspondence weakens, what weakens with it is not the resemblance of outcomes (a wrapped building and a moulted crustacean both end up with a new outer layer) but the location of the agency that produced them.

Across all four modes, the building is acted upon. In nature, the equivalent modes are performed by the skin upon itself, and the capacity to perform them is present before the need arises. It is this second condition, rather than any particular technique, that current practice does not reproduce.

The mapping is also not one-to-one, and its asymmetries are as informative as its correspondences.

Regeneration appears as a counterpart to three of the four interventions. In natural skin, regeneration is a single process: lost or damaged tissue is rebuilt by the organism from its own resources. Buildings have no equivalent single process, and what stands in its place is a set of techniques, adding material, substituting material, covering over, and wrapping around, each reproducing some part of the outcome by a different route. The multiplicity on the architectural side corresponds to an absence rather than to a richness. Where natural skin has one capacity, practice has four workarounds.

Communication has no counterpart among the interventions at all. This is not because envelopes do not communicate; they have done so from cave painting to the media façade, and Kunsthaus Graz enters the case set on precisely that basis. It is because communication does not appear on the architectural side as a way of intervening. It appears as a reason for intervening. A façade may be replaced or wrapped in order to change what a building signifies (e.g., the Reichstag cupola was rebuilt as much for what it would mean as for what it would do), but the operation performed remains replacement or wrapping. What is a mode of action in nature is a motive for action in architecture.

Taken with the gradient described above, the two asymmetries indicate the same thing from a different direction. In natural skin, fitting modes are capacities the tissue possesses. In the building envelope, they are operations performed upon it. The correspondence breaks down at exactly the points where possession and performance cannot be made to align.

### 3.4. Coding of the Case Set

The fifteen cases were coded against the eight variables defined in Section 2.3. The results are given in Table 3.

Six patterns emerge.

The operational cases cluster at the shortest time scales. Every one of the eleven cases in Groups A and B is coded T1 or T1–T2 on the intended scale; none is designed to reach T3, and none is designed to address the building’s life cycle. This holds across a span of nearly four decades, from the Institut du Monde Arabe (1987) to Solar Gate (2024), and across every actuation principle represented in the set: motorised, pneumatic, material-embedded, and living. The primary prediction registered in Section 2.4 is confirmed without exception.

Fitting modes cluster on protection. Of the eleven cases in Groups A and B, ten are coded P; the single exception, Kunsthaus Graz, is coded C. Regeneration and growth do not appear in Groups A and B at all; instead, they are confined to the life-cycle cases of Group C, where change takes the form of dismantling and reuse. This reproduces, on an enlarged and re-coded set, the earlier finding that adaptive envelopes concentrate on energy and aesthetics [26]. The absence is not incidental: regeneration and growth are the two modes that operate over the life of an organism rather than over its daily cycles, and they are absent from precisely the group whose time coding is confined to T1 and T2.

Material-based capacity does not, on its own, make a case integrated. Of the cases whose adaptive capacity resides in the material itself or in a living medium (BIQ House, Homeostatic Façade, HygroSkin, Flectofin, Solar Gate) only HygroSkin is coded INT. In the remainder, the removal test returns a functioning envelope: the photobioreactor panels of the BIQ House are mounted on a conventional façade; the dielectric elastomer strips of the Homeostatic Façade occupy the cavity of a double-skin unit; Flectofin is a component designed for attachment; and Solar Gate’s self-shaping elements are installed within box windows that would remain serviceable without them. Fourteen of the fifteen cases in the set are coded SUB.

The Homeostatic Façade System illustrates how far material-based capacity can be from material-driven behaviour. Its dielectric elastomer ribbons deform through a property of the polymer, which places the case under MAT, but the deformation is produced by an electrical charge distributed across a silver electrode layer and regulated by an actuator, which places the stimulus under E. The material is doing the work, and the material is being told when to do it. Coding capacity type separately from stimulus keeps this visible, and it distinguishes the case from HygroSkin and Solar Gate, where no supply and no control are involved.

This distinction is only visible because removability and capacity type were coded as separate variables. A single locus variable would have collapsed them, and the finding, that an envelope can be biomimetic at the level of its material while remaining an addition at the level of its system, would have been lost.

Cases that reach T4 do so externally, and none has been tested. Four cases are coded T4: BUGA Wood Pavilion, Triodos Bank, Circle House, and the module of Niazy et al. All are coded EXT or depend on external agency for the life-cycle provision. In each, the capacity for long-term change consists of provisions that allow others to take the building apart: dry connections, mechanical fixings, catalogued components, a material passport, or a re-orientable joint.

In no case has the anticipated change yet occurred. The Triodos building stands as constructed. The Circle House housing scheme has not been built; a full-scale demonstrator was completed in 2020, but the sixty-unit project remains at tender stage, as recorded in the architects’ own project documentation. The reuse of the BUGA Wood Pavilion’s components was announced as an intention and could not be confirmed from a reliable source. The intended time-scale column of Table 3 records design intent, while the observed column records what has occurred, and for these four cases it is empty.

This is the reason the two were coded as separate variables. Demountability is documented here as a property of design rather than as an outcome, and the distinction is material: a provision for change that has not been exercised is not yet evidence that the provision works.

**Figure 3:**
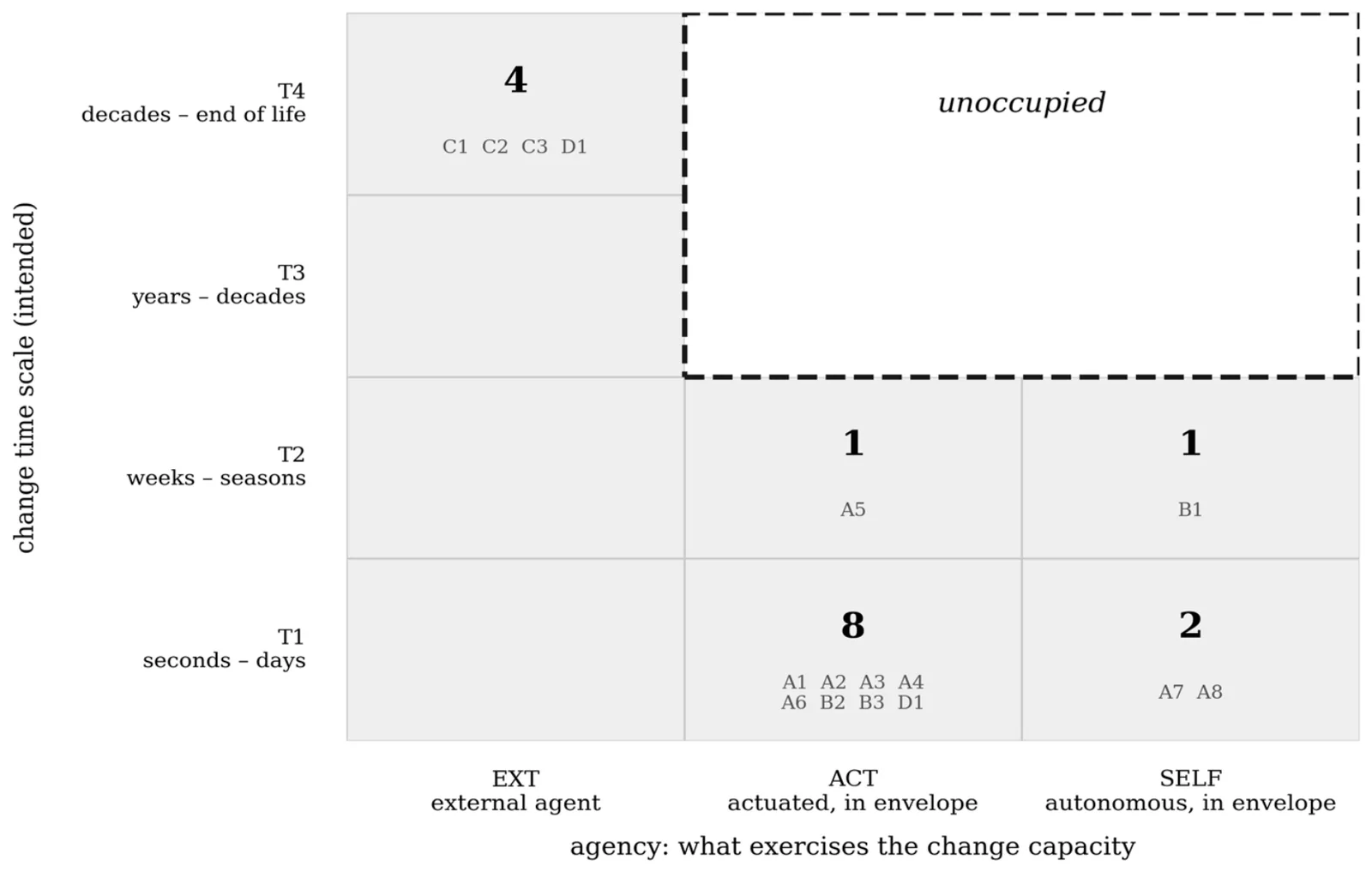
Distribution of the fifteen cases across intended change time scale (vertical) and agency—what exercises the change capacity (horizontal): EXT, external agent; ACT, actuated within the envelope; SELF, autonomous within the envelope. Case identifiers follow Table 3; D1 appears in two cells because it engineers an operational (T1) and a life-cycle (T4) provision separately. The dashed region marks the combination that is unoccupied in this set: no case accommodates life-cycle change (T3–T4) through a capacity held within the envelope, whether autonomous or actuated. The only case coded INT on the separate removability axis (HygroSkin, A7) sits at T1.

No case combines an integrated, autonomous capacity with a life-cycle time scale (Figure 3). Separating the dimensions that the earlier scheme had combined makes the result stronger rather than weaker because it now holds on two independent axes. On the removability axis, the only case coded INT (HygroSkin) operates at T1. On the agency axis, every case whose intended scale is T4 (Triodos Bank, Circle House, the BUGA Pavilion, and the life-cycle half of the Niazy module) locates its capacity in an external agent (EXT), while every case whose capacity is autonomous or actuated (SELF or ACT) operates at T1–T2. The empty intersection therefore does not depend on collapsing removability and agency into one variable. Instead, it is visible when they are coded separately, which is evidence that it is not an artefact of the coding structure. Distinguishing intended from observed time scale sharpens rather than dissolves the point. The absence is an absence of systems intended to accommodate T3–T4 change through the envelope’s own capacity; it is not an absence of T3-scale events. The Institut du Monde Arabe reached T3 as an observed outcome, but as a failure, and its recovery came from outside the envelope. On the interpretation set out in Section 4.3, this is a finding for the present set and a hypothesis for the field.

Within the analysed set, the intermediate time scale is unoccupied on the intended-scale coding: no case is coded T3 as a design intent. The distribution divides between an operational cluster at T1–T2 and an end-of-life cluster at T4, with nothing designed for the interval between them. The one case that reaches T3—the Institut du Monde Arabe—does so only as an observed outcome and as a failure, not as an intended scale. This was not predicted, and it is the more striking absence. T3 is the scale at which buildings actually become unfit. Requirements drift, occupancies change, components reach the end of their service life, and envelopes are re-clad, re-glazed, or insulated—all of this occurs over years and decades, and none of it is addressed by any case examined here.

Neither literature claims the scale. It is too slow for adaptive façade research, whose methods resolve behaviour over cycles of hours and seasons, and too early for end-of-life planning, whose provisions are exercised when the building is taken apart. The Institut du Monde Arabe failed at exactly this scale, and its failure is legible only in retrospect because no framework was watching there.

#### 3.4.1. Two Cases That Qualify the Pattern

Two cases warrant separate comment because they mark the limits of what the field has achieved.

Solar Gate is the strongest instance of a material-based, autonomous capacity in the set. Its 424 shading elements are 4D-printed from bio-based hygroscopic material and change shape in response to humidity and temperature without any electrical supply, sensor, or control system. The system was tested under real weather conditions for over a year before installation on the south-facing skylight of a permanent research building. It succeeds by any measure of the biomimetic ambition, including the capacity residing in the material, the absence of external energy, and the robustness demonstrated in service.

It is coded T1–T2. The daily and seasonal cycles it responds to are the cycles it was designed for, and the question of how the assembly will be maintained, replaced, or adapted over the building’s life is outside its scope. Solar Gate demonstrates that a material-based, autonomous capacity is achievable in a real building; it does not demonstrate that such a capacity has been extended beyond the operational scale.

The Institut du Monde Arabe is the only case in the set with a fully documented long-term outcome, and that outcome is instructive. The 240 motorised diaphragms of the south façade required substantial maintenance, degraded progressively, and ceased operation in the early 2000s, after roughly fifteen years, leaving the façade static [27]. The design intent was T1; the diaphragms ceased to perform at T3. What is telling is not only the failure but its remedy: the mechanism was not restored by any capacity within the envelope, but by a major external intervention fifteen years later. For the building’s thirtieth anniversary in 2017, the façade was renovated in full, with the broken parts replaced, the mechanism re-engineered, and a new control system installed, at a cost of some 3.5 million euros funded by donation [46]. Long-term fitness, in other words, was recovered not from the organisation of the envelope but from outside it, after a fifteen-year interruption; the case is coded T1 for the behaviour it was designed to perform, and its life-cycle outcome belongs to external agency.

The failure is instructive because it was not a failure of the T1 design. The diaphragms performed the function they were designed for. What was not designed for was the maintenance burden, the component lifespan, and the institutional capacity to service a bespoke mechanism over decades—none of which are properties of the adaptive behaviour itself, and none of which appear in the performance criteria by which such systems are evaluated. An envelope can satisfy every criterion at the scale it addresses and still cease to fit at a scale it does not.

#### 3.4.2. Where the Envelope Sits in Each Group

A further pattern appears when the groups are compared on what part of the building carries the provision for change.

In Groups A and B, the envelope is the subject of the work. In Group C, it is not. The demountable element of the Triodos Bank is its timber structure, joined with screws rather than adhesives, and registered in a material passport. The façade is a different matter: it is a curtain wall carried on a steel structure, and the project documentation of the client, architect, and façade contractor reports that the question of how to make it circular was taken up separately, after completion, including the possibility of transferring the façade to a consortium under a service contract [39]. That arrangement corresponds to the façade-as-a-service model examined in the circular construction literature [44,45], where responsibility for an envelope is retained by its supplier rather than transferred with the building. In Circle House, the six standardised demountable elements are all structural: two wall types, two beam lengths, and two deck lengths. The envelope is not among them.

The same observation held for the two cases considered and excluded during case selection: a modular car park with a demountability index had almost no envelope to assess, and a business park designed as a material bank did not resolve to a single envelope as a unit of analysis.

The two literatures therefore fail in complementary ways. The biomimetic literature takes the envelope as its object but confines itself to operational time scales. The life-cycle literature reaches the life-cycle scale but locates the provision for change in the structure, where components are large, few, repetitive, and mechanically separable—the conditions under which design for disassembly is most tractable. The envelope, which is layered, bonded, weather-critical, and composed of dissimilar materials in intimate contact, is the part of the building that resists those conditions most strongly.

The empty cell is thus empty from both directions and for different reasons on each side.

## 4. Discussion

### 4.1. From Retrofitting to Adaptive Fitting

The coding returns a set of results that are individually unsurprising and jointly difficult to reconcile. Envelopes that adapt do so within hours or seasons. Envelopes that anticipate change over decades do so by making themselves easy to take apart. No case does both, and no case locates the capacity for long-term change anywhere other than in an external agent. The Institut du Monde Arabe shows what this costs: a façade that satisfied its operational criteria and ceased to function within a few years, defeated by a time scale its design did not address.

Taken together, these results suggest that the two literatures are not addressing the same problem at different scales but are addressing different problems. The adaptive façade literature asks how an envelope should behave. The disassembly literature asks how an envelope should end. Neither asks how an envelope should remain fit, which is the question that the mapping in Section 3.2 raises. In natural skin, the modes by which fitting is maintained over an organism’s life are the same modes that operate day to day, and they are performed by the skin on itself.

The term adaptive fitting is used here for the design approach that follows from taking this seriously, and it is worth separating from two neighbouring terms. The fitting modes (Section 3.1) are the biological processes by which a natural skin keeps itself fit; retrofitting is what a building receives once it has ceased to fit; and adaptive fitting is the specification, during early design, of the conditions under which a building can continue to fit. Its object is not any particular future change but the process of fitting itself. Four characteristics follow from the findings above.

#### 4.1.1. Fitting Arises from Within Rather than by External Addition

Fourteen of the fifteen cases returned a working envelope when their adaptive capacity was removed. The adaptive layer performs the adaptive function, and the rest of the envelope is unaffected by it. This is the condition that the removal test was designed to detect, and it is close to universal in the set.

The exception is instructive. HygroSkin returns no functioning envelope when its capacity is removed because there is nothing to remove. The responsive behaviour is a property of the material and its geometry, specified at the point of design. Solar Gate operates on the same principle under real weather, although as an insert within a conventional opening. Neither requires an actuator, a sensor, or a control system; the responsive capacity is programmed into the material itself rather than supplied by equipment [34].

Designing for adaptive fitting means locating the capacity for change in the envelope’s own material and geometric organisation, so that later modification draws on provisions already specified rather than on provisions brought to the site. The decision this implies is earlier than the decisions normally understood as adaptive design. What is specified is not a mechanism but the conditions under which change becomes possible without one.

#### 4.1.2. The Prior Condition Constructs the Subsequent One

In the four modes of intervention examined in Section 3.2, the existing fabric is the object of the operation. Material is added to it, removed from it, or wrapped around it, and in each case its own properties are incidental to what follows. Wrapping is the limit case: it preserves the old envelope precisely by rendering it inert.

Natural fitting proceeds differently. Growth at an edge, moulting, and regeneration all build the new condition out of the old one, and the form of the result is constrained by what was already there. An envelope designed for adaptive fitting would carry the information required to produce its next state without determining what that state will be. This distinguishes the approach both from scenario planning, which fixes the alternatives in advance, and from flexibility understood as surplus capacity, which supplies room without supplying direction.

This is the characteristic for which the case set offers no instance, and it is stated here as a proposition rather than a finding. Its plausibility rests on the analogy; its demonstration would require a design.

#### 4.1.3. Capacity Is Specified Across Time Scales Together

One case bridges the two clusters, and it is worth emphasising because it shows the intersection is not unreachable in principle. The separation between the operational cluster and the life-cycle cluster is otherwise complete: no case appears in both except the Niazy module, which engineers each scale separately. Niazy et al. combined an operational capacity based on servo motors with a reuse capacity based on demountable spherical joints, achieving adaptability at both scales by adding a distinct provision for each. Their own discussion identifies the difficulty this creates, noting that a more durable motor would be needed to reduce the module’s annual maintenance, the same problem that removed the Institut du Monde Arabe’s façade from service, anticipated at the prototype stage.

The distinction between responding to a summer afternoon and responding to a change of use is one of duration, not of kind. In natural skin, both are served by the same organisation. Adaptive fitting requires that the two be considered together at the point of design, so that provisions made for one are not made at the expense of the other. This is the characteristic that most sharply separates the approach from current practice, where the two scales are handled by different specialists, at different project stages, under different criteria, and where the coupling between them becomes visible only as failure.

#### 4.1.4. Fitting Requires Distinguishing What Changes from What Does Not

Natural systems conserve older organisation while accommodating new adaptation, and the boundary between the two is not arbitrary. The equivalent design act is to identify which parts of the envelope are expected to absorb change and which are expected to hold, and to detail them differently on that basis.

Of the four characteristics, this is the most immediately operational, and it is also the one on which the others depend. Without it, capacity for change is either withheld where it is needed or distributed everywhere at cost. It is the characteristic that current practice comes closest to, and the comparison with design for disassembly is therefore worth making precisely.

#### 4.1.5. Relation to Adjacent Approaches

Adaptive fitting is not design for disassembly, and it is not the combination of adaptive and circular design proposed by Niazy et al. The three can be distinguished by where they locate the capacity for change.

Design for disassembly locates it in the connections. Dry fixings, catalogued components, and material passports allow a building to be taken apart efficiently by others, and the flagship cases in this set (Triodos Bank, Circle House) demonstrate how far this can be carried. Two observations qualify it. First, in both cases, the provision is made in the structure. For example, the Triodos façade is a curtain wall whose circularity was addressed separately, after completion. Second, disassembly of contemporary façade systems is not standard end-of-life practice [16,18], and none of the cases coded T4 here has yet undergone the change it was designed for. The approach is well developed as design method and largely undemonstrated as outcome.

The approach of Niazy et al. adds an operational capacity to a demountable one within a single module, and this is the only case in the set to address both time scales. Both capacities remain mechanical and both remain additions: the module is attached to a conventional opening, and its removal returns a working envelope.

Adaptive fitting differs from both in locating the capacity for change in the envelope itself, at both time scales. It is compatible with disassembly and does not exclude mechanisms. What it proposes is that these should not be the primary means by which an envelope remains fit.

#### 4.1.6. Two Boundaries

First, adaptive fitting is a design approach, not a construction technique. It does not prescribe materials, joints, or assemblies. The question of how its principles are to be embodied belongs to the emulation phase of biomimetic design and is not addressed here.

Second, the approach is untested. The findings reported above establish that the intersection of an integrated, autonomous capacity and a life-cycle time scale is unoccupied; they do not establish that it can be occupied. Solar Gate demonstrates that a material-based, autonomous capacity is achievable in a building at operational scale, which makes the extension plausible, but plausibility is not demonstration. On this point adaptive fitting stands where design for disassembly stands: a design proposition whose long-term claims have not yet been exercised.

### 4.2. Why the Intersection Remains Unoccupied

The unoccupied region in Figure 3 could be interpreted as neglect, reflecting a combination that no one has happened to try. The coding suggests otherwise. Three conditions, each visible in the results, work to keep it empty.

The envelope is the least tractable part of the building for disassembly. The provision for long-term change in both Group C cases is made in the structure. Triodos Bank’s demountability resides in a timber frame assembled with screws rather than adhesives, and Circle House reduces its structure to six repeating precast elements. Structures of this kind offer the conditions under which design for disassembly works best: components that are large, few, repetitive, mechanically separable, and not required to be continuous. Envelopes offer none of these. They are layered, frequently bonded, composed of dissimilar materials in intimate contact, and required to remain weather-tight across every junction. Where the two are addressed in the same project, the structure is where the effort goes, and the Triodos façade, whose circularity was taken up separately and after completion, shows what happens to the envelope when it is addressed second.

Adaptive envelope research validates at the scale it can test. The biomimetic cases in the set are evaluated by simulation, by prototype testing, or by field measurement over months. Solar Gate, the most thoroughly validated case, was tested under real weather for more than a year before installation. These methods can establish how an envelope behaves across a diurnal or seasonal cycle. They cannot establish how it behaves across a change of use or three decades of maintenance because the relevant events are neither simulatable nor observable within a research cycle. A field that validates in this way will tend to ask questions it can answer.

The performance criteria do not include the adaptive system’s own persistence. This is the condition the Institut du Monde Arabe makes visible. It is the most consequential of the three, and it accounts for the empty T3 row. The diaphragms were assessed, as adaptive envelopes generally are, on thermal and visual performance during operation. Nothing in that assessment concerned the maintenance burden of 240 bespoke mechanisms, the service life of their components, or the institutional capacity required to keep them working. Those factors determined the outcome. An envelope can meet every criterion by which it is judged and still cease to fit, because the criteria describe its behaviour and not its persistence.

The same omission appears at the other end of the set. Niazy et al. identified motor durability and annual maintenance as matters for future work, having designed a module whose reuse capacity depends on those motors continuing to function. The problem is recognised and deferred, which is not a criticism of that study specifically, as no established evaluation framework would have required it to be resolved. What is deferred in both cases belongs to the same scale, the years and decades over which a mechanism must keep working, and it is deferred because no party to the design is asked about it.

If the omission is one of criteria, the constructive question is what criteria would close it. The present study does not resolve this, but the pattern it reports points to the kind of measure that is missing. Adaptive envelopes are evaluated on instantaneous or seasonal performance, thermal gain, daylight, energy, and not on the persistence of the adaptive capacity itself. Three candidate measures would begin to address that gap. They are offered as proposals to be tested rather than as a validated framework: a mean time between failures for the adaptive mechanism, analogous to the reliability metrics used in engineered systems; a maintenance-access provision, recording whether the components that will require servicing can be reached and replaced without disturbing the weather envelope; and a replacement-cycle predictability, recording whether the service lives of the adaptive components are known and coordinated with the shearing layers of the building. None of these is developed or applied here; each would require the kind of longitudinal or engineering study that lies outside the scope of this paper, and their development is one of the directions set out below. Their purpose in this argument is narrower: to show that the empty T3 row corresponds to an absence in the evaluation criteria, and that the absence is in principle remediable.

Taken together, these conditions suggest that the gap is not a capability gap. A material-based, autonomous capacity has been achieved in a building: Solar Gate operates without electrical supply, sensors or controls, and its capacity is inseparable from its material. What has not been attempted is the extension of that principle to the scale at which buildings actually become unfit. This suggests, as a hypothesis rather than a demonstrated cause, that the obstacle is not an inability to build such a capacity but an absence of anything in the way envelopes are commissioned, designed, evaluated, or researched that directs attention to the time scale at which the question arises.

This also indicates where the proposition advanced here is most vulnerable and where it would first need testing. If adaptive fitting is to be more than a reframing, it must produce design decisions that differ from those that current criteria would produce, and it must be assessable by criteria that current practice does not apply. The second requirement may prove harder than the first.

### 4.3. Scope and Limitations

The claims of this study are bounded in three ways and are made only within those bounds. First, the evidence is a purposive set of fifteen cases, not a systematic review. It was chosen to test whether a combination appears in the field’s most prominent and best-documented work, not to measure how often it occurs. The empty cell, combining a life-cycle (T3–T4) intended scale with an integrated, autonomous capacity, is therefore a finding for this set and a hypothesis for the field. It shows that the combination is absent from these strong cases, not that the field contains none. The set is also institutionally and regionally concentrated, and no case coded at life-cycle scale records a realised change. Instead, those codings rest on design intent, as the empty observed-scale column shows. Second, the coding is interpretive and was carried out by one researcher, without independent inter-coder agreement. The removability test, in particular, is applied by a single criterion and is contestable in cases such as the BIQ House. To offset this, the scheme, the definitions, and the per-case sources are given in full so that the coding can be independently re-applied and disputed, representing a form of reproducibility that does not replace multi-coder validation but keeps the interpretive steps visible. Third, the biological material is used analogically and at a level of generality. The four fitting modes are a working taxonomy that organises the comparison, not a claim about the mechanism in any organism, and not the only possible partition. What the analysis can do is classify these cases and generate hypotheses; what it cannot do is establish that the field as a whole lacks such approaches, or that adaptive fitting is the right response. Those are questions for the systematic and longitudinal work this study is meant to motivate. As a check on how far the central result depends on contestable coding, the most debatable assignments were re-coded under two plausible alternatives: the BIQ House as INT rather than SUB, and the actuated cases (ACT) grouped with the external-agency cases rather than with the autonomous ones. Under each alternative, the cases with a T4 intended scale still carry no integrated, autonomous capacity, so the empty intersection is not an artefact of these particular decisions. This is a sensitivity check, not a substitute for independent inter-coder validation, which remains the appropriate next step.

Two cases considered during selection and set aside—a modular multi-storey car park with a published demountability index and a business park designed as a material bank—bear on this. Both sit at the life-cycle end of the range and both locate their provision for change in the structure rather than the envelope, so that including them would have reinforced rather than challenged the central finding. They would have added further cases at T4 and EXT without occupying the intersection. They were excluded because neither resolves to a building envelope as a unit of analysis (Section 3.4.2), not because their codings would have unsettled the pattern.

## 5. Conclusions

This study set out to determine whether the separation between adaptive façade research and design for disassembly reflects a real division in the problem of keeping building envelopes fit or a division of academic labour. The evidence assembled here supports the second interpretation.

Four modes of intervention through which envelopes are currently kept fit (add-on, replace, cover it, and wrap it) were identified and mapped onto four fitting modes of natural skin. The correspondence weakens as the intervention becomes more external, and in all four, the building is acted upon rather than acting. Fifteen documented cases were then coded against a scheme separating the time scale at which change is accommodated from the location and type of capacity accommodating it.

The coding produced three findings. Every biomimetic envelope in the set operates at daily or seasonal scale, across nearly four decades of practice and every actuation principle represented. Fourteen of fifteen cases return a functioning envelope when their adaptive capacity is removed, including cases whose capacity resides in the material—biomimetic material behaviour does not by itself produce integration. And every case addressing life-cycle change does so through external agency, with no case having yet undergone the change it anticipates.

Two absences follow. No case is coded at the intermediate scale of years to decades at all. The set divides between an operational cluster and an end-of-life cluster, with nothing between them, although this is the scale at which buildings in fact become unfit. And the combination of an integrated, autonomous capacity with a life-cycle time scale does not occur. This combination has been termed adaptive fitting and is characterised as the specification, during early design, of the conditions under which a building can continue to fit: capacity arising from the envelope’s own organisation rather than from additions to it; the prior condition constructing the subsequent one; the two time scales specified together; and an explicit distinction between what is expected to change and what is expected to hold.

The most consequential result is not the empty cell but the account of why it stays empty. A material-based, autonomous capacity is not beyond the field’s reach, as Solar Gate achieves it in a permanent building, without electrical supply, sensors, or controls. What is missing is any reason for the field to extend it. Envelopes are evaluated on how they behave, not on how long they continue to behave that way. The maintenance burden, component life, and institutional capacity that determined the fate of the Institut du Monde Arabe appear in no standard assessment of adaptive envelope performance, and the same omission is visible in current prototype work. This motivates a hypothesis, which the observed pattern supports but does not by itself establish, that the obstacle is one of evaluation before it is one of capability. Confirming it would require evidence beyond the case pattern, on design requirements, commissioning practice, maintenance criteria, and research incentives. This is left to future work; here, it is offered as an interpretation of the pattern, not as a demonstrated cause.

Two directions follow. The first is the emulation phase of biomimetic design: establishing what material and geometric organisation could carry change capacity at life-cycle scale, which this study has framed but not attempted. The second is prior to it, and may be the more urgent: developing assessment criteria under which the persistence of an adaptive envelope, and not only its performance, becomes a question that design is required to answer.

These two directions also fix the boundary of what the present study claims. The engineering questions that adaptive fitting eventually raises are real and, in the end, decisive for practice: how it is realised in curtain-wall, modular or double-skin systems, how it satisfies structural, fire and weather-tightness requirements across successive adaptations, how it integrates with building services, and how it performs under life-cycle assessment and life-cycle costing. They are not answered here, and answering them is premature. A design approach must first be specified, and criteria for judging its persistence must first exist before it can be validated against them. The contribution of this paper is analogical and diagnostic rather than technical. Its value, if it has one, is in reframing the question from how an envelope should behave, or how it should end, to how it should remain fit, and not in supplying a solution. The engineering that would follow from taking the reframing seriously is the substance of the work that comes after.

Adaptive fitting remains a proposition. Its long-term claims are untested, as are those of the approaches it is set against. What the evidence supports is narrower, and arguably sufficient to be worth stating: the envelope has been asked to adapt and asked to come apart, and it has not yet been asked to remain fit.

## Figures and Tables

**Table 1:** Coding scheme.

Variable	Status	Codes
Scale of change	retained	WE, whole envelope; PE, parts or volumes; LEE, larger element; SEE, small elements
Fitting mode	retained	R, regeneration; G, growth; P, protection; C, communication
Stimulus source	retained	E, environmental (external) stimulus—light, heat, humidity, wind; I, internal stimulus—a building-system signal or schedule. Records the source that triggers the response, not the location of the capacity; a dash (—) is entered where the case has no operational response.
Change time scale (intended)	new	T1, seconds–days; T2, weeks–seasons; T3, years–decades; T4, decades–end of life. The scale the design is intended to accommodate
Change time scale (observed)	new	The scale at which a change or failure has actually been documented; a dash (—) is entered where none is reported
Removability	new	INT, removal leaves no functioning envelope (integrated); SUB, removal leaves a functioning envelope (substitutable)
Capacity type	new	MAT, material behaviour; LIV, living medium (a cultured organism, e.g., microalgae); MEC, discrete mechanism; a dash (—) is entered where the provision is not a material or mechanism but a disassembly protocol
Agency	new	SELF, capacity operates autonomously, without supplied energy; ACT, capacity is actuated by supplied energy (a motor, pump or electrical charge); EXT, capacity is exercised by an external agent (a contractor or facilities team)
Validation status	new	BUILT, in service; PROTO, prototype, pavilion or component; SIM, simulation only

**Table 2:** Fitting modes of natural skin and modes of intervention in the building envelope.

Fitting Mode of Natural Skin	Strategies in Nature	Mode of Intervention in the Envelope	Typical Operation
Regeneration	Renewal; restoration	Add-on	New structure attached; existing fabric passive
Growth	Growth at an edge; growth with shells; moulting; addition	Cover it	Existing space enclosed; footprint unchanged
Growth/regeneration	Shell exchange; moulting	Replace	Elements substituted; structure retained
Protection/regeneration	Strengthening; colouration	Wrap it	Second layer applied over existing envelope
Communication	Signalling; sexual attraction	no counterpart (see Section 3.3)	A motive for intervening rather than a mode of intervening

**Table 3:** Coding of the case set (*n* = 15).

#	Case	Scale	Mode	Remov.	Cap.	Agency	Stim.	T-int.	T-obs.	Valid.	Source
A1	Institut du Monde Arabe (1987)	SEE	P	SUB	MEC	ACT	E	T1	T3	BUILT	[27]
A2	Al Bahar Towers (2012)	SEE	P	SUB	MEC	ACT	E	T1	—	BUILT	[27,28]
A3	Media-TIC (2011)	PE	P	SUB	MEC	ACT	E	T1	—	BUILT	[27,29]
A4	Kunsthaus Graz (2003)	WE	C	SUB	MEC	ACT	E	T1	—	BUILT	[30]
A5	BIQ House (2013)	WE	P	SUB	LIV	ACT	E	T1–T2	—	BUILT	[31]
A6	Homeostatic Façade (2011)	PE	P	SUB	MAT	ACT	E	T1	—	PROTO	[32]
A7	HygroSkin (2013)	PE	P	INT	MAT	SELF	E	T1	—	PROTO	[33,34]
A8	Flectofin (2011)	PE	P	SUB	MAT	SELF	E	T1	—	PROTO	[35]
B1	Solar Gate, livMatS (2024)	SEE	P	SUB	MAT	SELF	E	T1–T2	—	BUILT	[11]
B2	FlectoLine (2024)	PE	P	SUB	MEC/MAT	ACT	E	T1	—	BUILT	[36,37]
B3	Mimosa kinetic (2023)	PE	P	SUB	MEC	ACT	E	T1	—	SIM	[12]
C1	Triodos Bank HQ (2019)	WE	G/R	SUB	—	EXT	—	T4	—	BUILT	[39,44,45]
C2	Circle House, Lisbjerg (2023)	WE	G/R	SUB	—	EXT	—	T4	—	PROTO	[40]
C3	BUGA Wood Pavilion (2019)	WE	G	SUB	—	EXT	—	T4	—	BUILT	[38]
D1	Circular adaptive module (2023)	PE	P	SUB	MEC	ACT/EXT	E	T1+T4	—	PROTO	[21]

Notes to Table 3. The Source column gives the reference(s) from which each case was coded. Two time-scale columns are given: T-int. is the scale the design is intended to accommodate, and T-obs. is the scale at which a change or failure has actually been documented (a dash where none is reported). No case has a realised life-cycle change; the four cases with a T4 intended scale are coded on the provision made for change, not on a change that has occurred. Triodos Bank (C1): the demountable structure and material passport are in place but not yet exercised. Circle House (C2): the sixty-unit residential project remains at tender stage, a full-scale demonstrator was completed in 2020, and the case is treated as a pilot. BUGA Wood Pavilion (C3): component reuse was announced as an intention but could not be confirmed from a reliable source. Circular adaptive module (D1): a laboratory prototype whose reuse capacity has not been tested in service. Removability is applied by a single criterion—whether removing the adaptive provision leaves a weather-tight, load-bearing envelope. The BIQ House (A5) is coded SUB on this basis, because its photobioreactor panels are mounted on a conventional façade that remains functional without them, although a performance-based reading (thermal, visual, energy) would qualify this and is noted in Section 4.3. A dash under Capacity or Stimulus marks a life-cycle case with no material or mechanism locus and no operational response. Codes (defined in full in Table 1): Scale—WE, whole envelope; PE, parts or volumes; LEE, larger element; SEE, small elements. Mode—R, regeneration; G, growth; P, protection; C, communication. Removability—INT, integrated; SUB, substitutable. Capacity—MAT, material; LIV, living medium; MEC, mechanism. Agency—SELF, autonomous; ACT, actuated; EXT, external agent. Stimulus source—E, environmental; I, internal. Validation—BUILT, constructed and in service; PROTO, prototype; SIM, simulation.

## Data Availability

All data supporting the reported findings are contained within the article.
